# Synthesis, Characterization, Antimicrobial, DNA Cleavage, and Antioxidant Studies of Some Metal Complexes Derived from Schiff Base Containing Indole and Quinoline Moieties

**DOI:** 10.1155/2013/315972

**Published:** 2013-10-01

**Authors:** Mahendra Raj Karekal, Vivekanand Biradar, Mruthyunjayaswamy Bennikallu Hire Mathada

**Affiliations:** Department of Studies and Research in Chemistry, Gulbarga University, Gulbarga-585 106, Karnataka, India

## Abstract

A new Schiff base of 5-chloro-3-phenyl-1*H*-indole-2-carboxyhydrazide and 3-formyl-2-hydroxy-1*H*-quinoline (**HL**), and its Cu(II), Co(II), Ni(II), Zn(II), Cd(II), and Hg(II) complexes have been synthesized and characterized in the light of microanalytical, IR, H1 NMR, UV-Vis, FAB-mass, ESR, XRD, and TGA spectral studies. The magnetic susceptibility measurements and low conductivity data provide evidence for monomeric and neutral nature of the complexes. On the basis of spectral studies and analytical data, it is evident that the Schiff base acts as tridentate ligand. The Cu(II), Co(II), and Ni(II) complexes were octahedral, whereas Zn(II), Cd(II), and Hg(II) complexes were tetrahedral in nature. The redox behavior of the Cu(II) complex was investigated by electrochemical method using cyclic voltammetry. In order to evaluate the effect of metal ions upon chelation, both the ligand and its metal complexes were screened for their antibacterial and antifungal activities by minimum inhibitory concentration (MIC) method. The DNA cleavage experiment performed using agarose gel electrophoresis method showed the cleavage of DNA by all the metal complexes. The free radical scavenging activity of newly synthesized compounds has been determined at a different concentration range by means of their interaction with the stable free radical 1,1-diphenyl-2-picrylhydrazyl (DPPH).

## 1. Introduction

Numerous indole-containing natural and synthetic products such as reserpine, vincristine, indolemycin, mitomycin, pindolol, dolasetron mesylate, indomethacin, or sumatriptan are being used for the treatment of various illnesses. Therefore, indole structure represents a highly relevant heterocyclic system. Many pharmacodynamic compounds containing indole nucleus have been reported to possess a wide variety of biological properties, namely, anti-inflammatory [1, 2], anticonvulsant [3], cardiovascular [4], antibacterial [5], COX-2 inhibitor [6, 7], and antiviral activities [8]. More specifically, several reports describe that indole-2-carbohydrazides and related compounds are endowed with antihistaminic [9], antidepressant [10], and MAO inhibitory activities [11]. Particularly, the compounds having three substituted indole nucleus are being used as the starting materials for the synthesis of number of alkaloids, agrochemicals, pharmaceuticals, and perfumes [12].

Quinoline derivatives have also attracted the attention of the chemists because of their presence in many natural products possessing significant biological activities [13–17]. Small-molecule interactions with DNA continue to be intensely and widely studied for their usefulness as probes of cellular replication and transcriptional regulation and for their potential as pharmaceuticals. The Cu(II) complexes have been reported to be active in DNA strand scissors [18]. On the other hand, an increasing interest in antioxidant, particularly in those intended to prevent the mischievous effects caused by the free radicals in the human body is an attracting one. The free radicals are also believed to be associated with carcinogenesis, mutagenesis, arthritis, diabetes, inflammation, cancer, and genotoxicity due to the oxidative stress which arises as a result of imbalance between free radical generations [19, 20]. The antioxidant activity of metal complex is found to be induced by both the identity of the metal and the ligands bound to it [21]. The development of new metal-containing antioxidant agents have been reported by several research groups [22]. Based on these findings and in continuation of our research work on coordination chemistry [23–28], we describe the synthesis of a new Schiff base and its metal complexes with 5-chloro-3-phenyl-1*H*-indole-2-carboxyhydrazide fused to 3-formyl-2-hydroxy-1*H*-quinoline with the aim of obtaining more potent pharmacological active compounds. 

## 2. Experimental

### 2.1. Analysis and Physical Measurements

IR Spectra of the synthesized Schiff base and its metal complexes were recorded in KBr pellets on a Perkin-Elmer FT-IR instrument in the range 4000–350 cm^−1^. ^1^H NMR spectra were recorded in *d*_6_-DMSO using a Bruker DRX-400 MHz instrument. UV-Visible spectra of the Cu(II), Co(II), and Ni(II) complexes were recorded on Elico-SL 164 spectrometer in the range 200–1000 nm in DMF solution (1 × 10^−3^ M). The FAB mass spectra of ligand and its Cu(II) and Zn(II) complexes were recorded on a JEOL SX 102/DA-6000 mass spectrometer/data system using argon/xenon (6 kV, 10 mA) as the FAB gas. The accelerating voltage was 10 kV and the spectra were recorded at room temperature using m-nitrobenzyl alcohol (NBA) as the matrix. Elemental analysis was obtained from HERAEUS C, H, and N–O rapid analyzer, and metal analysis was carried out by following the standard methods. ESR measurement was carried out on a BRUKER BioSpin Gmbh spectrometer working at microwave frequency of 9.903 GHz. Electrochemistry of the Cu(II) complex was recorded on a 600 D series model electrochemical analyzer in DMF using *n*-Bu_4_N–ClO_4_ as a supporting electrolyte. The experiment was carried out by using DPPH as reference with field set at 3200 gauss. Magnetic susceptibility measurements were made at room temperature on a Gouy balance using Hg[Co(NCS)_4_] as the calibrant. 

### 2.2. Methods

All the chemicals used were of reagent grade and procured from Hi-media and Sigma Aldrich. Solvents were dried and distilled before use. Melting points of the newly synthesized compounds were determined by electrothermal apparatus using open capillary tubes. The metal and chloride contents were determined as per standard procedures [29]. The precursors 5-chloro-3-phenyl-1*H*-indole-2-carboxyhydrazide and 3-formyl-2-hydroxy-1*H*-quinoline were prepared by the literature methods [16, 30].

### 2.3. Synthesis of the Schiff Base **HL**

**Scheme 1:**
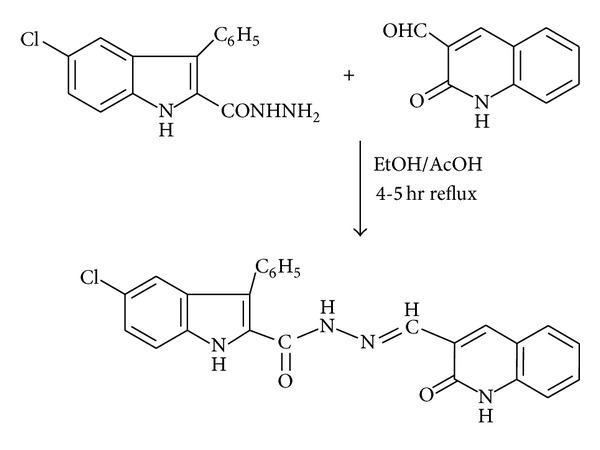
Synthesis of Schiff base ligand HL.

Equimolar mixture of 5-chloro-3-phenyl-1*H*-indole-2-carboxyhydrazide (0.001 mol) and 3-formyl- 2-hydroxy-1*H*-quinoline (0.001 mol) with a catalytic amount of glacial acetic acid (1-2 drops) in ethanol (20 mL) was refluxed on a water bath for about 7-8 h. The reaction was monitored by TLC. The pale yellow solid separated was filtered, washed with little ethanol, dried, and recrystallized from dioxane (Scheme 1). Yield: 65%; m.p. 314°C. Anal. Calcd. for C_25_H_17_O_2_N_4_Cl (Mr = 440): C, 68.10; H, 3.85; N, 12.71%. Found: C, 68.25; H, 3.91; N, 12.89%. IR (KBr, cm^−1^): 3311, 3229, and 3162 (*ν*_NH/NH_); 1674 and 1661 (*ν*_C=O_); 1608 (*ν*_C=N_); ^1^H NMR (DMSO-*d*_6_): *δ* 12.20 and 12.00 (s, 2H, two CONH); 11.60 (s, 1H, indole NH); 8.40 (s, 1H, HC=N); 7.10–8.20 (m, 13H, ArH). 

### 2.4. Preparation of Cu(II), Co(II), Ni(II), Zn(II), Cd(II), and Hg(II) Complexes of Schiff Base **HL**

To the hot solution of 5-chloro-*N*-(2′-dihydro-2′-oxoquinolin-3′-yl methylene)-3-phenyl-1*H-*indole-2-carbohydrazide (HL) (0.002 mol) in ethanol (30 mL) was added a hot ethanolic solution (15 mL) of respective metal chlorides (0.002 mol). The reaction mixture was refluxed on a steam bath for about 4 h, during which no solid separated out. An aqueous alcoholic solution of sodium acetate (0.5 g) was added to the reaction mixture to maintain a pH of about 6.0–7.0 and reflux was continued for about an hour. The reaction mixture was poured in the distilled water. The separated solid complexes were collected by filtration, washed with sufficient quantity of distilled water, then with hot ethanol to apparent dryness, and dried in a vacuum over anhydrous calcium chloride in a desiccator. The melting points of all the compounds are reported in Table 1.

#### 2.4.1. Cu(II) Complex of Schiff Base HL

*Green Solid*. Yield: 70%; m.p. > 340°C; Anal. Calcd. for [Cu(C_50_H_32_O_4_N_8_Cl_2_)]H_2_O (Mr = 959.54): C, 62.46; H, 3.53; N, 11.66%. Found: C, 62.59; H, 3.32; N, 11.74%. IR (KBr, cm^−1^): 3418 (*ν*_H_2_O_); 3283 and 3221 (*ν*_NH/NH_); 1639 (*ν*_C=O_); 1548 (*ν*_C=N_); 1493, 1077 and 615 (*ν*_Pyridine  ring_); 514 (*ν*_M–O_); 442 (*ν*_M–N_). UV-Vis (cm^−1^): *ν*_2_, 13769–17463.

#### 2.4.2. Co(II) Complex of Schiff Base HL

*Light Brown Solid*. Yield: 77%; m.p. > 340°C; Anal. Calcd. for [Co(C_50_H_32_O_4_N_8_Cl_2_)]H_2_O (Mr = 954.93): C, 62.76; H, 3.55; N, 11.71%. Found: C, 62.92; H, 3.82; N, 11.86%. IR (KBr, cm^−1^): 3423 (*ν*_H_2_O_); 3271 and 3230 (*ν*_NH/NH_); 1629 (*ν*_C=O_); 1551 (*ν*_C=N_); 1489, 1061 and 670 (*ν*_Pyridine  ring_); 553 (*ν*_M–O_); 435 (*ν*_M–N_). UV-Vis (cm^−1^): *ν*_1_, 7447; *ν*_2_, 15977; *ν*_3_, 19518.

#### 2.4.3. Ni(II) Complex of Schiff Base HL

*Brown Solid*. Yield: 79%; m.p. > 340°C; Anal. Calcd. for [Ni(C_50_H_32_O_4_N_8_Cl_2_)]H_2_O (Mr = 954.69): C, 62.78; H, 3.55; N, 11.71%. Found: C, 62.92; H, 3.69; N, 11.91%. IR (KBr, cm^−1^): 3430 (*ν*_H_2_O_); 3305 and 3218 (*ν*_NH/NH_); 1638 (*ν*_C=O_); 1555 (*ν*_C=N_); 1478, 1063 and 621 (*ν*_Pyridine  ring_); 477 (*ν*_M–O_); 450 (*ν*_M–N_). (FAB+) MS: M^•+^ 955, 957, 959 (10%, 6%, 3%); *m/z* 937, 939, 941 (25%, 50%, 70%); 498, 500 (50%, 30%); 244 (18%). Uv-Vis (cm^−1^): *ν*_1_, 9680; *ν*_2_, 15604; *ν*_3_, 25634.

#### 2.4.4. Zn(II) Complex of Schiff Base HL

*Light Yellow Solid*. Yield: 69%; m.p. > 340°C; Anal. Calcd. for [Zn(C_25_H_16_O_2_N_4_Cl)(Cl)]H_2_O (Mr = 557.40): C, 53.72; H, 3.22; N, 10.02%. Found: C, 53.99; H, 3.35; N, 10.19%. IR (KBr, cm^−1^): 3420 (*ν*_H_2_O_); 3305 and 3218 (*ν*_NH/NH_); 1634 (*ν*_C=O_); 1557 (*ν*_C=N_); 1489, 1066 and 615 (*ν*_Pyridine  ring_); 470 (*ν*_M–O_); 425 (*ν*_M–N_); 357 (*ν*_M–Cl_). ^1^H NMR (DMSO-*d*_6_): *δ* 13.12 (s, 1H, CONH); 11.53 (s, 1H, indole NH); 8.45 (s, 1H, HC=N); 7.35–8.55 (m, 12H, ArH). (FAB+) MS: M^•+^ 557, 559, 561 (10%, 18%, 15%); *m/z* 539, 541, and 543 (48%, 72%, 20%); 469 (48%); 250 (60%). 

#### 2.4.5. Cd(II) Complex of Schiff Base HL

*Pale Yellow Solid*. Yield: 71%; m.p. > 340°C; Anal. Calcd. for [Cd(C_25_H_16_O_2_N_4_Cl)(Cl)]H_2_O (Mr = 604.41): C, 49.55; H, 2.97; N, 9.24%. Found: C, 49.71; H, 2.81; N, 9.35%. IR (KBr, cm^−1^): 3423 (*ν*_H_2_O_); 3293 and 3221 (*ν*_NH/NH_); 1637 (*ν*_C=O_); 1550 (*ν*_C=N_); 1425, 1059, and 699 (*ν*_Pyridine  ring_); 469 (*ν*_M–O_); 419 (*ν*_M–N_); 350 (*ν*_M–Cl_). ^1^H NMR (DMSO-*d*_6_): *δ* 13.14 (s, 1H, CONH); 11.62 (s, 1H, indole NH); 8.46 (s, 1H, HC=N); 7.20–8.35 (m, 12H, ArH).

#### 2.4.6. Hg(II) Complex of Schiff Base HL

*Light Yellow Solid*. Yield: 68%; m.p. 330°C; Anal. Calcd. for [Hg(C_25_H_16_O_2_N_4_Cl)(Cl)] (Mr = 674.59): C, 44.40; H, 2.36; N, 8.28%. Found: C, 44.61; H, 2.42; N, 8.36%. IR (KBr, cm^−1^): 3295 and 3230 (*ν*_NH/NH_); 1664 (*ν*_C=O_); 1560 (*ν*_C=N_); 1420, 1060, and 640 (*ν*_Pyridine  ring_); 550 (*ν*_M–O_); 428 (*ν*_M–N_); 352 (*ν*_M–Cl_). 

### 2.5. Pharmacology

#### 2.5.1. Antimicrobial Assays

The biological activities of the synthesized Schiff base **HL** and its Cu(II), Co(II), Ni(II), Zn(II), Cd(II), and Hg(II) complexes were studied for their antibacterial and antifungal activities by the disc and well diffusion method, respectively. The *in vitro* antibacterial activities of the compounds were tested against two Gram-negative *Escherichia coli *(MTCC 46) and *Salmonella typhi* (MTCC 98) and two Gram-positive* Bacillus subtilis* (MTCC 736) and* Staphylococcus aureus* (MTCC 3160) bacteria. The *in vitro* antifungal activities were carried out against* Candida albicans *(MTCC 227),* Cladosporium oxysporum *(MTCC 1777), and* Aspergillus niger *(MTCC 1881) fungi [31, 32]. The stock solutions of the test chemicals (1 mg mL^−1^) were prepared by dissolving 10 mg of the each test compound in 10 mL of distilled DMSO solvent. The different concentrations of the test compounds (100, 75, 50, 25, and 12.5 *μ*g mL^−1^) were prepared by diluting the stock solution with the required amount of freshly distilled DMSO. Further the controlled experiments were carried out by using freshly distilled DMSO solvent alone.

#### 2.5.2. Antibacterial Screening

Muller-Hinton agar media was used for the antibacterial studies. The pure dehydrated Muller-Hilton agar (38 g) was dissolved in 1000 mL distilled water. The pure cultures of the bacterial strains* E. coli*, *S. aureus*,* B. subtilis*, and *S. typhi *were subcultured by inoculating in the nutrient broth, and they were incubated at 37°C for about 18 h. The agar plates were prepared by using the above Muller-Hinton agar media, and wells were dug with the help of 6 mm sterile metallic cork borer. Each plate was inoculated with 18-h-old bacterial culture (100 *μ*L) using a micropipette and spreaded uniformly using bent glass rod on each plate. The drug gentamycin is used as standard. Different concentration of the test compounds were incorporated into the wells using micropipette, and the plates were kept for incubation at 37°C for 24 h. Soon after the completion of incubation period, the diameter of the inhibition zone generated by each test compound against bacterial growth was measured using antibiogram zone measuring scale.

#### 2.5.3. Antifungal Screening

Potato dextrose agar (PDA) media was used for the antifungal studies. The following ingredients were used to prepare the media: potatoes (sliced washed unpeeled) 200 g, dextrose 20 g, and agar 20 g in 1000 mL distilled water. The pure cultures* C. albicans*,* C. oxysporum*, and* A. niger *were inoculated on PDA slants. These slants were incubated at 32°C for 7 days. To these 7-day-old slants of fungal strains, 10 mL of 0.1% tween-80 solution was added, and the culture were scraped with sterile inoculating loop to get uniform spore suspension. The agar plates were prepared by using the above potato dextrose agar media and wells were dug with the help of 6 mm sterile metallic cork borer. Each plate was inoculated with 7-day-old spore suspension of each fungal culture (100 *μ*L) using a micropipette and spreaded uniformly using bent glass rod on each plate. Then each well was incorporated with the test compound solution of different concentrations. The drug fluconazole is used as standard. All the inoculated plates were incubated at 32°C for about 48 h. Soon after the completion of incubation period the diameter of the inhibition zone generated by each test compound against fungal growth was measured using antibiogram zone measuring scale.

#### 2.5.4. DNA Cleavage Experiment

The extent to which the newly synthesized ligands and their metal complexes could function as DNA cleavage agents was examined using *E. coli* DNA as a target. The electrophoresis method was employed to study the efficiency of cleavage by the synthesized compounds. Nutrient broth media was used (Peptone 10 g, NaCl 10 g and yeast extract 5 gL^−1^) for culturing *E. coli*. The electrophoresis of the test compounds was done according to the literature method [33]. 

The freshly prepared *E. coli* culture (1.5 mL) is centrifuged, and the pellets obtained, which was then dissolved in 0.5 mL of lysis buffer (50 mM EDTA, 100 mM tris pH 8.0, 50 mM lysozyme). To this, 0.5 mL of saturated phenol was added and incubated at 55°C for 10 min. Soon after the incubation the solution was centrifuged at 10,000 rpm for 10 min, and to the supernatant liquid, equal volume of chloroform: isoamyl alcohol (24 : 1) and 1/20th volume of 3 M sodium acetate (pH 4.8) were added. Again the solution is centrifuged at 10,000 rpm for 10 min and the supernatant layer collected is then mixed with 3 volumes of chilled absolute alcohol, and the DNA precipitates. The precipitated DNA was separated by centrifugation, and the pellet was dried and dissolved in Tris buffer (10 mM tris pH 8.0) and stored in cold condition.

Agarose (250 mg) was dissolved in hot tris-acetate-EDTA (TAE) buffer (25 mL) (4.84 g Tris base, pH-8.0, 0.5 M EDTA L^−1^), and heated to boil for few minutes. When the gel attains approximately 55°C, it was then poured into the gas cassette fitted with comb. Slowly the gel was allowed to solidify by cooling to room temperature and then carefully the comb was removed. The solidified gel was placed in the electrophoresis chamber containing TAE buffer. Test compounds (1 mg mL^−1^) were prepared in DMSO. The test compounds (25 *μ*g) were added to the isolated DNA of *E. coli*, and they were incubated for 2 h at 37°C. Soon after the incubation period the DNA sample (20 *μ*L) mixed with bromophenol blue dye in equimolar ratio along with standard DNA marker containing TAE buffer was loaded carefully into the wells, and the constant 50 V of electricity was supplied for about 30 min. Later, the gel was removed, and it was stained with ethidium bromide solution (10 *μ*g mL^−1^) for 15–20 min, then the bands were observed and photographed under UV- illuminator.

#### 2.5.5. Antioxidant Assay (Free Radical Scavenging Activity)

The free radical scavenging activity of the test samples was determined with the 2,2-diphenyl-1-picryl-hydrazyl (DPPH) method [34]. Different concentrations of test compounds (10 *μ*g, 50 *μ*g, and 100 *μ*g) and standard butylated hydroxy anisole (BHA) were taken in different test tubes, and the volume of each test tube was adjusted to 100 *μ*L by adding distilled DMF. To the sample solution in DMF, 5 mL methanolic solution of DPPH (0.1 mM) was added to these tubes. The tubes were allowed to stand for 30 min. The control experiment was carried out as above without the test samples. The absorbance of test solutions was measured at 517 nm. The reduction of DPPH was calculated relative to the measured absorbance of the control. Radical scavenging activity was calculated using the following formula:

(1) %  Radical  scavenging  activity=[Control  optical  density−Sample  optical  densityControl  optical  density]×100.

## 3. Results and Discussion

All the synthesized metal complexes are coloured solids, amorphous in nature, and stable in air. Melting points of the newly synthesized metal complex were above >300°C. The complexes are insoluble in water and common organic solvents but are soluble in solvents like DMF and DMSO. Elemental analysis and analytical data of the complexes suggest that the metal to ligand ratio of the complexes is 1 : 2 stoichiometry of the type [M(L)_2_]H_2_O for Cu(II), Co(II), and Ni(II) complexes, 1 : 1 stoichiometry of the type [M(L)(Cl)]H_2_O for Zn(II) and Cd(II) complexes and [M(L)(Cl)] for Hg(II) complex, respectively, where L stands for deprotonated ligand. The molar conductance values are too low to account for any dissociation of the complexes in DMF (24–60 ohm^−1^ cm^2^ mole^−1^), indicating their nonelectrolytic nature of the complexes in DMF (Table 1) [35]. 

### 3.1. IR Spectral Studies

The important IR bands of the Schiff base **HL** and its metal complexes are represented in Table 2. IR spectrum of Schiff base **HL** displayed one sharp band at 3311 and two weak bands at 3229 and 3162 cm^−1^ due to indole NH and two NH/NH functions of two amide groups, respectively. Two sharp peaks observed at 1674 and 1661 cm^−1^ are due to two carbonyl groups of two CONH functions. A band at 1608 cm^−1^ is due to C=N of azomethine group of the Schiff base. Disappearance of band at 1674 cm^−1^ due to quinolinone carbonyl function with the appearance of a new band in the region 1290–1287 cm^−1^ in all the complexes proves the enolization of quinolinone carbonyl during complexation and formation of a new bond between enolized oxygen and metal ion via deprotonation. The shift of band due to amide carbonyl attached to 2-position of indole from 1661 cm^−1^ to 1639–1629 cm^−1^ in case of all the complexes confirms the coordination of metal ion with oxygen atom of this carbonyl function without undergoing enolization [36]. The bands appeared at 3305–3283 and 3230–3218 cm^−1^ due to indole NH and NH of amide function attached to 2-position of indole, respectively, in case of complexes it proves the noninvolvement of these NH functions in complexation, since these two peaks have appeared at 3311 and 3229 cm^−1^ in case of Schiff base **HL**. Absorption frequency of HC=N function which has appeared at 1608 cm^−1^ in the Schiff base has been shifted towards lower frequency by 60–48 cm^−1^ and appeared in the region 1568–1548 cm^−1^ in all the complexes which indicates the coordination of metal ion with azomethine nitrogen atom [37]. This is further confirmed by the appearance of new bands in the region 555–469 cm^−1^ and 470–419 cm^−1^ in all the complexes which are due to *ν*_M–O_ and *ν*_M–N_ stretching vibrations, respectively [38]. In Zn(II), Cd(II), and Hg(II) complexes of ligand **HL**, new bands observed in the region 357–350 cm^−1^ are due to *ν*_M–Cl_ vibrations.

Bands observed in the region 1493–1420, 1077–1059, and 699–615 cm^−1^ in all the complexes are due to pyridine ring vibration of quinoline moiety. The broad band observed in all the complexes except Hg(II) complex of ligand **HL** in the region 3430–3418 cm^−1^ is assigned to *ν*_OH_ vibration of the lattice water molecules [39].

### 3.2. ^1^H NMR Spectral Studies

The ^1^H NMR data of Schiff base **HL** and its Zn(II) and Cd(II) complexes are presented in Table 3. The ^1^H NMR spectrum of Schiff base **HL** displayed three broad singlets at 12.20, 12.00, and 11.60 ppm which are due to proton of two amide functions (s, 2H, CONH) and proton of indole NH(s, 1H, NH), respectively. The signals due to proton of azomethine function and thirteen aromatic protons have appeared at 8.40 ppm (s, 1H, HC=N) and 7.10–8.20 ppm (m, 13H, ArH), respectively.

In the spectra of Zn(II) and Cd(II) complexes displayed two peaks at 13.12, 11.53 ppm and 13.14, 11.62 ppm due to proton of an amide function and proton of indole NH, respectively. The disappearance of the signal at 12.00 ppm which was there in the spectrum of Schiff base **HL** confirms the enolization of CONH function of quinolinone moiety and its involvement in complexation with metal ion through oxygen atom of enolized carbonyl function via deprotonation. The signal due to azomethine proton in Zn(II) and Cd(II) complexes have been shifted towards lower field strength when compared to the spectra of ligand; they have appeared at 8.45 and 8.46 ppm, respectively. The aromatic protons in case of Zn(II) and Cd(II) complex have been resonated in the region 7.35–8.55 and 7.20–8.35 ppm, respectively. The shift of the entire proton towards lower field strength when compared to Schiff base **HL** proves beyond doubt the formation of complex with metal ion. 

### 3.3. FAB-Mass Spectral Studies

**Figure 1:**
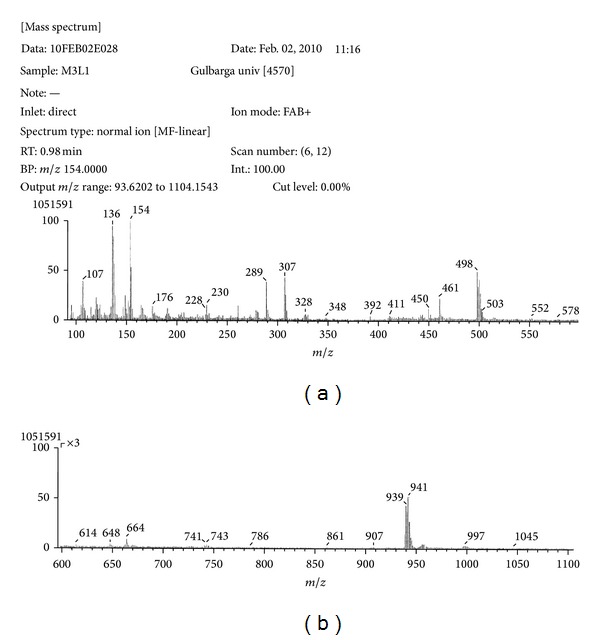
FAB-mass spectrum of Ni(II) complex.

**Scheme 2:**
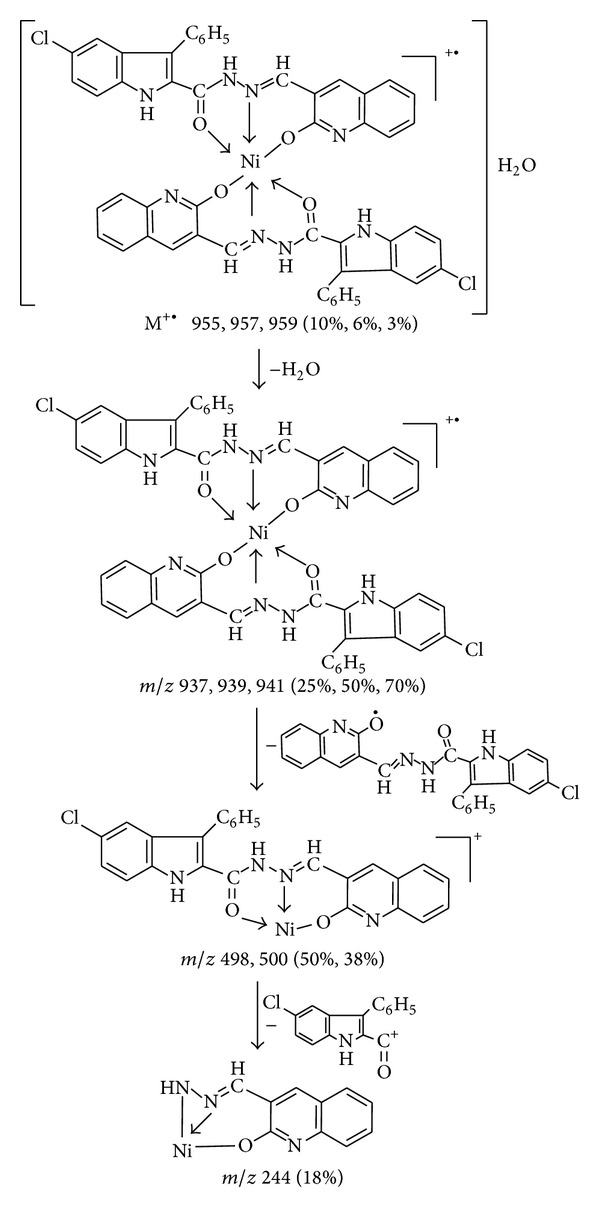
Mass fragmentation of Ni(II) complex.

The representative Ni(II) and Zn(II) complexes have been subjected for their mass spectral studies. The FAB-mass spectrum of Ni(II) complex is depicted in Figure 1, shows a molecular ion peak at M^•+^ 955, 957, and 959 (2%, 6%, 18%) which corresponds to its molecular weight, confirming the stoichiometry ratio of metal chelates as [M(L)_2_]H_2_O. Further fragment ion observed at *m/z* 937, 939, 941 (25%, 50%, 70%), 498, 500 (50%, 30%), and 244 (20%) are due to the sequential expulsion of H_2_O, C_25_H_16_N_3_O_2_Cl, and C_15_H_9_NOCl species, respectively, from the molecular ion. This fragmentation pattern is in consistency with its structure (Scheme 2). 

**Figure 2:**
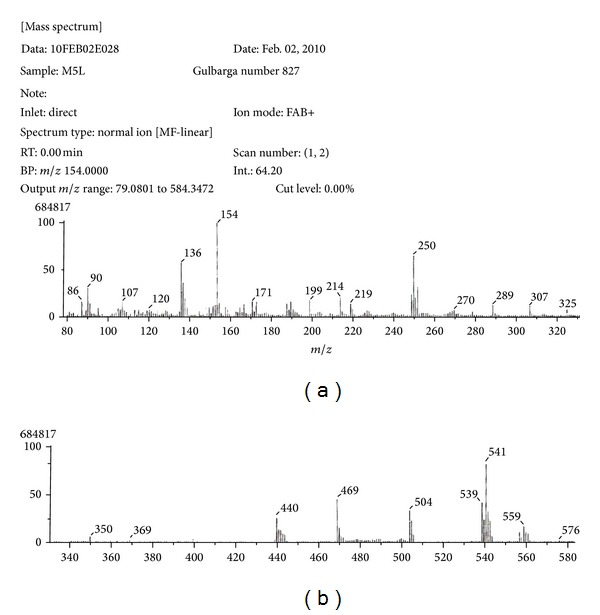
FAB-mass spectrum of Zn(II) complex.

**Scheme 3:**
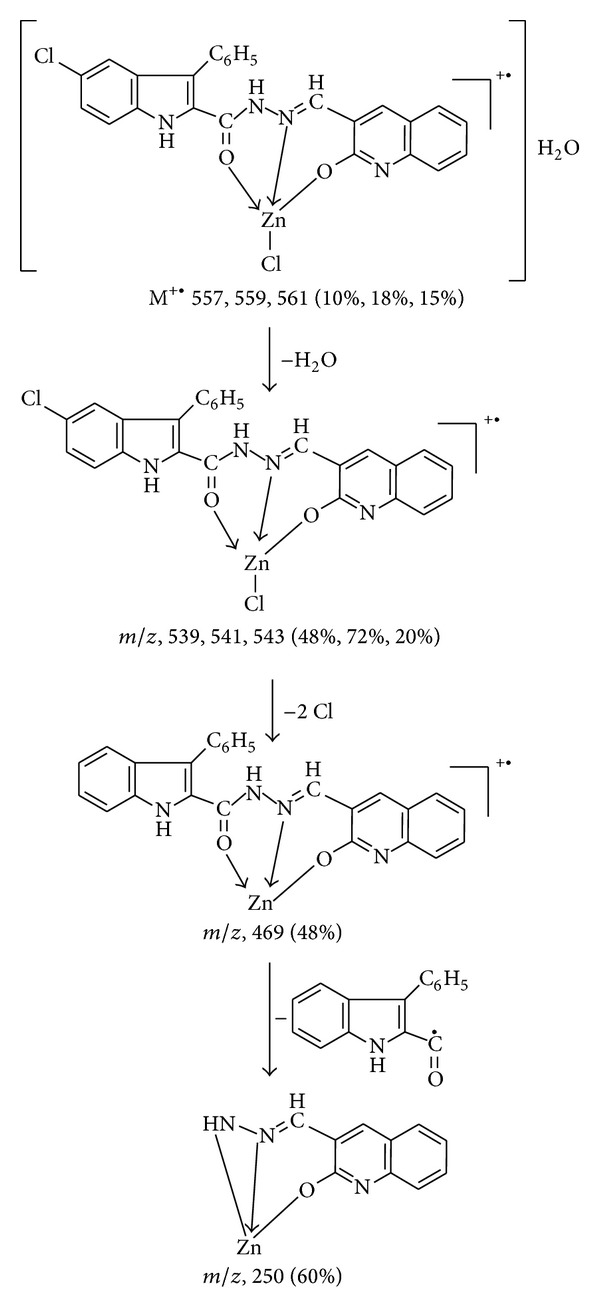
Mass fragmentation of Zn(II) complex.

The FAB-mass spectrum of Zn(II) complex (Figure 2) shows a molecular ion peak M^•+^ 557, 559, and 561 (10%, 18%, 14%) which corresponds to its molecular weight, confirming the stoichiometry ratio of metal chelates as [M(L)(Cl)]H_2_O. Further fragment ion observed at *m/z* 539, 541, 543 (48%, 72%, 20%), 469 (48%), and 250 (58%) are due to the sequential expulsion of H_2_O, 2Cl, and C_15_H_9_NO species, respectively, from the molecular ion. This fragmentation pattern (Scheme 3) is in conformity with the structure.

### 3.4. Electronic Spectral Studies

Electronic spectral data of the Cu(II), Co(II), and Ni(II) complexes of the Schiff base **HL** are given in Table 4. Electronic spectral studies of all these complexes were carried out in DMF at 10^−3^ M concentration. The green coloured Cu(II) complex displayed low intensity single broad band in the region 13769–17463 cm^−1^. The broadness of the band is assigned due to ^2^B_1g_ → ^2^Eg, ^2^B_1g_ → ^2^B_2g_ and ^2^B_1g_ → ^2^A_tg_ transitions, which are similar in energy and give rise to only one broad absorption band, and the broadness of the band is due to dynamic Jahn-Teller distortion. These data suggest that the Cu(II) complex have distorted octahedral geometry [40]. 

The electronic spectra of brown coloured Co(II) complex shows two absorption bands observed at 15977 cm^−1^ and 19518 cm^−1^ due to the ^4^T_1g_  (F) → ^4^A_2g_  (F) (*ν*_2_) and ^4^T_1g_  (F) → ^4^T_2g_(P) (*ν*_3_) transitions, respectively, which are in good agreement with the reported values [41]. The lowest band, *ν*_1_ is not to be observed due to the limited range of the instrument used but could be calculated using the band fitting procedure suggested by Underhill and Billing [42]. These transitions suggest octahedral geometry for the Co(II) complex. 

The brown Ni(II) complex under present investigation exhibited two bands in the region 15604 cm^−1^ and 25634 cm^−1^. These bands are assigned to ^3^A_2g_  (F) → ^3^T_1g_  (F) (*ν*_2_) and ^3^A_2g_  (F) → ^3^T_1g_  (P) (*ν*_3_) transitions, respectively, in an octahedral environment. The band *ν*_1_ was calculated by using a band fitting procedure [42].

The octahedral geometry was further supported by the values of ligand field parameters, such as the Racah interelectronic repulsion parameter (*B*′), ligand field splitting energy (10 Dq), nephelauxetic parameter (**β**), and ligand field stabilization energy (LFSE) [43]. The calculated *B*′ values for the Co(II) and Ni(II) complexes were lower than the free ion values, which is due to the orbital overlap and delocalisation of d-orbitals. The **β** values are important in determining the covalency for the metal-ligand bond, and they were found to be less than unity, suggesting a considerable amount of covalency for the metal-ligand bonds. The **β** value for the Ni(II) complex was less than that of the Co(II) complex, indicating the greater covalency of the M–L bond [44].

### 3.5. Magnetic Susceptibility Studies

Magnetic susceptibility measurements obtained at room temperature for Cu(II), Co(II), and Ni(II) complexes are listed in Table 1, and they were found to be paramagnetic in nature. The observed magnetic moment for Cu(II) complex is 1.79 BM. The observed value is slightly higher than the spin-only value due to one unpaired electron 1.73 BM suggesting the octahedral geometry [45]. Thus, the present Cu(II) complex is devoid of any spin interaction with distorted octahedral geometry. In octahedral Co(II) complex the ground state is ^4^T_1g_. A large orbital contribution to the singlet state lowers the magnetic moment values for the various Co(II) complexes which are in the range 4.12–4.70 and 4.70–5.20 BM for tetrahedral and octahedral complexes, respectively [46]. In the present investigation the observed magnetic moment value for Co(II) complex is 5.01 BM which indicates octahedral geometry for the Co(II) complex. For Ni(II) complex the observed magnetic moment value is 2.90 BM which is well within the expected range for Ni(II) complex with octahedral stereochemistry 2.83–3.50 BM [47]. 

### 3.6. ESR Spectral Studies of the Cu(II) Complex

To obtain the information about the hyperfine and superhyperfine structure in order to elucidate the geometry of the complex and the site of the metal-ligand bonding or environment around the metal ion, the X-band ESR spectra of Cu(II) complex has been recorded in the polycrystalline state at room temperature at a frequency of 9.387 GHz with a field set of 3950 G. The spin Hamiltonian parameters for the Cu(II) complex is used to derive the ground state. In octahedral geometry the *g*-tensor parameter is with *g*_||_ > *g*_⊥_ > 2.0023, the unpaired electron lies in the d_*x*2-*y*2_ orbital in ground state and with *g*_⊥_ >*g*_||_ > 2.0023, and the unpaired electron lies in the d_*z*_^2^ orbital [48]. The observed measurements for Cu(II) complex, *g*_||_ (2.075) > *g*_⊥_ (2.018) > 2.0023 indicating that the complex is axially symmetric and copper site, has a d_*x*2-*y*2_ ground state characteristic of octahedral geometry [49]. The *g*_||_ value is an important function for indicating the metal-ligand bond character, for ionic *g*_||_ > 2.3 and for covalent characters *g*_||_ < 2.3, respectively [50]. In the present Cu(II) complex, the *g*_||_ value is less than 2.3, indicating an appreciable covalent character for the metal-ligand bond. The geometric parameter (*G*) is the measure of extent of exchange interaction and is calculated by using *g*-tensor values by the expression *G* = *g*_||_ − 2/*g*_⊥_ − 2. According to Hathaway and Billing [51], if the *G* value is greater than 4, the exchange interaction between the copper centers is negligible, whereas its value is less than 4 and the exchange interaction is noticed. The calculated *G*-value for the present Cu(II) complex is 4.166 indicating that the exchange coupling effects are not operative in the present complex.

### 3.7. Thermal Studies

The thermal stabilities of the complexes have been studied as a function of temperature. The proposed thermal decomposition pattern with temperature and the percentage of metal oxide obtained are depicted in Table 5.

**Figure 3:**
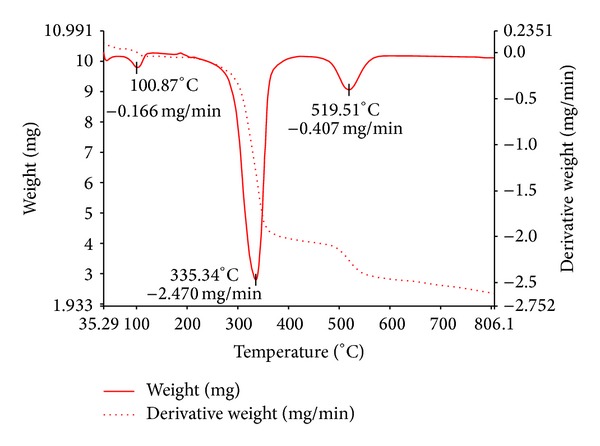
TG-DTA curve of Cu(II) complex.

The Thermo gravimetric curve (TGA) of the representative Cu(II) complex is shown in Figure 3, showing three decomposition steps. The first decomposition occurs at a temperature of 100°C which is due to the loss of a lattice water molecule, the second decomposition occurs at a temperature of 335°C which corresponds to loss of two C_15_H_10_N_3_OCl species, and the third decomposition occurs at a temperature of 519°C due to the loss of two C_4_H_4_ species. Further the complex underwent decomposition in a gradual manner rather than with the sharp decomposition up to 750°C corresponds to loss of remaining organic moiety, leaving behind the residue copper oxide.

The thermal decomposition pattern of Co(II), Zn(II), and Hg(II) complexes shows two decomposition steps each. The first decomposition occurs at a temperature of 100, 110, and 330°C which corresponds to loss of lattice water molecule with weight loss of 1.88% (Cald 1.59%) for Co(II) complex and 3.09% (Cald 3.22%) for Zn(II) complex, and the loss of HCl for Hg(II) complex with weight loss of 5.31% (Cald 5.33%), respectively. The second decomposition occurs at temperatures of 348, 340, and 354°C which correspond to the loss of two C_15_H_10_N_3_OCl species for Co(II) complex with weight loss of 61.55% (Cald. 59.47%), loss of C_15_H_10_N_2_OCl species for Zn(II) complex with a weight loss of 48.15% (Cald. 48.25%), and loss of C_19_H_13_ONCl species for Hg(II) complex with a weight loss of 49.46% (48.46%), respectively. Further, these complexes underwent gradual decomposition up to 750–850°C, corresponds to the loss of remaining organic moiety, leaving behind residue metal oxides of the respective complexes. The percentage metal composition in all the complexes as done by the elemental analysis agrees well with the remaining residual metal oxides in thermal studies.

### 3.8. Powder X-Ray Diffractions Studies

Although the synthesized metal complexes were soluble in some polar organic solvents (DMSO and DMF), crystals that are suitable for single-crystal studies are not obtained. Powder XRD pattern of Ni(II) and Zn(II) complexes are being studied in order to test the degree of crystallinity of the complexes. Powder X-ray diffraction pattern for Ni(II) complex showed 14 reflections in the range of 5–80° (2*θ*), which are arised from diffraction of X-ray by the planes of complex. The interplanar spacing (*d*) has been calculated by using Bragg's equation, *nλ* = 2*d*sin *θ*. The calculated interplaner *d*-spacing together with relative intensities with respect to most intense peak have been recorded and depicted in Table 6. The unit cell calculations have been calculated for cubic symmetry from the entire important peaks, and *h* ^2^ + *k* ^2^ + *l* ^2^ values were determined. The observed interplaner *d*-spacing values have been compared with the calculated ones, and it was found to be in good agreement. The *h* ^2^ + *k* ^2^ + *l* ^2^ values are 1, 3, 5, 6, 10, 15, 17, 39, 47, 83, 90, 109, and 138. It was observed that the presence of forbidden numbers such as 15 and 47 indicates that the Ni(II) complex may belong to hexagonal or tetragonal systems.

Similar calculations were performed for Zn(II) complex which showed 15 reflections in the range 5–80° (2*θ*). The important peaks have been indexed, and observed interplanar *d*-spacing values have been compared with the calculated ones. The unit cell calculations were performed for cubic system, and the *h* ^2^ + *k* ^2^ + *l* ^2^ values were determined. The values are 1, 2, 3, 4, 5, 7, 9, 11, 13, 22, 29, 41, 46, and 72. The presence of forbidden number such as 7 indicates the Zn(II) complex may belong to hexagonal or tetragonal systems (Table 7).

### 3.9. Electrochemical Study

**Figure 4:**
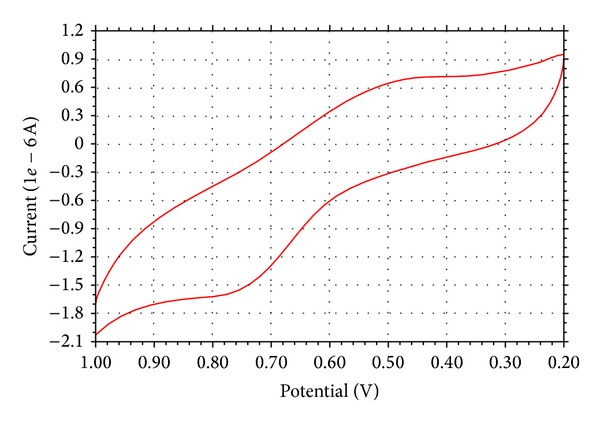
Cyclic voltammogram of Cu(II) complex at a scan rate of 100 mV/s.

Electron transfer plays a vital role in governing the pathway of chemical reactions. Cyclic voltammetry is the most versatile electroanalytical technique for the study of electroactive species. The electrochemical behaviour of Cu(II) complex was investigated in DMF (10^−3^ M) solution containing 0.05 M *n*-Bu_4_N–ClO_4_ as a supporting electrolyte by cyclic voltammetry, and it is the most versatile electroanalytical technique for the study of electroactive species. The cyclic voltammogram of Cu(II) complex (Figure 4) in DMF at a scan rate of 100 mV/s shows well-defined redox process corresponding to the formation of Cu(II)/Cu(I) couple at *E*_pa_ = 0.76 V and *E*_pc_ = 0.47 V versus Ag/AgCl. The peak separation of this couple is found to be quasi-reversible with Δ*E*_*p*_ = 0.29 V, and the ratio of anodic to cathodic peak height was less than one. The difference between forward and backward peak potential can provide a rough evaluation of the degree of the reversibility of one electron transfer reaction. Thus, the analysis of cyclic voltammetric response to 100 mV/s, 200 mV/s, and 300 mV/s scan rates gives the evidence for quasi-reversible one electron redox process. The ratio of anodic to cathodic peak height was less than one, and peak current increases with the increase of square root of the scan rates, establishing diffusion controlled electrode process [52]. From the peak separation value Δ*E*_*p*_ and peak potential increases with higher scan rates, we can suggest that the electrode processes are consistent with the quasi-reversibility of Cu(II)/Cu(I) couple [53].

### 3.10. Pharmacological Results

#### 3.10.1. *In Vitro* Antimicrobial Activity

The *in vitro* antimicrobial activity of all the synthesized compounds were screened against *E. coli, S. typhi, B. subtilis,* and* S. aureus* bacteria and* C. albicans, C. oxysporum, *and* A. niger *fungal strains by minimum inhibitory concentration (MIC) method. The minimum inhibitory concentration (MIC) profiles of all the compounds against bacteria and fungi are summarized in Table 8. The MIC values indicated that all the complexes exhibited promising results than the ligand against mentioned microorganisms, and this activity enhanced on coordination with the metal ions. This enhancement in the activity may be rationalized on the basis that ligands mainly possess C=N bond. The enhanced activity of the complexes over the ligand can be explained on the basis of chelation theory [54, 55]. It is observed that, in a complex, the positive charge of the metal is partially shared with the donor atoms present in the ligand, and there may be **π**-electron delocalization over the whole chelating [56]. This increases the lipophilic character of the metal chelate and favors its permeation through the lipoid layer of the bacterial membranes. The heterocyclic Schiff bases with different functional groups have greater tendency to interact with nucleoside bases even after complexation with metal ion or with the essential metal ions present in the biosystem can act as a promising bactericides because they always tend to interact with enzymatic functional groups, in order to achieve higher coordination numbers [57]. There are also other factors which increase the activity, namely, solubility, conductivity, and bond length between the metal and the ligand.

#### 3.10.2. DNA Cleavage Activity

**Figure 5:**
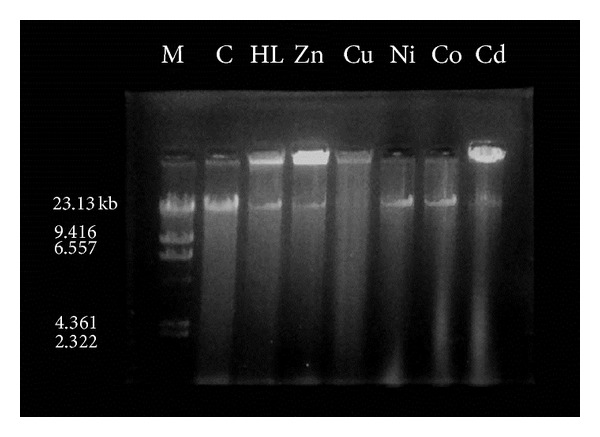
DNA cleavage of *E. coli *genome. M: standard molecular weight marker; C: control. Lane HL, Zn, Cu, Ni, Co, and Cd treated DNA of *E. coli* genome with respective compounds.

The representative Schiff base **HL** and its metal complexes are studied for their DNA cleavage activity by the agarose gel electrophoresis method against DNA of *E. coli*. The characterization of DNA recognition by transition metal complex has been aided by the DNA cleavage chemistry that is associated with redox-active or photoactivated metal complexes [58]. The electrophoresis analysis clearly revealed that the Schiff base and their metal complexes have acted on DNA as there was a difference in molecular weight between the control and the treated DNA samples. The difference was observed in the bands of lanes of complexes compared with the control DNA of *E. coli* (Figure 5) which is due to the relaxation of circular DNA into linear form. This shows that the control DNA alone does not show any apparent cleavage, whereas the Schiff base and its complexes do show. The lane Cu shows complete cleavage of DNA of *E. coli*, whereas the other complexes have shown partial cleavage. The result indicates the important role of coordination of nitrogen and oxygen to the metal in these isolated DNA cleavage reactions. As the compound was observed to cleave the DNA, it can be concluded that the compounds inhibit the growth of the pathogenic organism by cleaving the DNA of *E. coli*.

#### 3.10.3. Antioxidant Assay (DPPH Free Radical Scavenging Activity)

**Figure 6:**
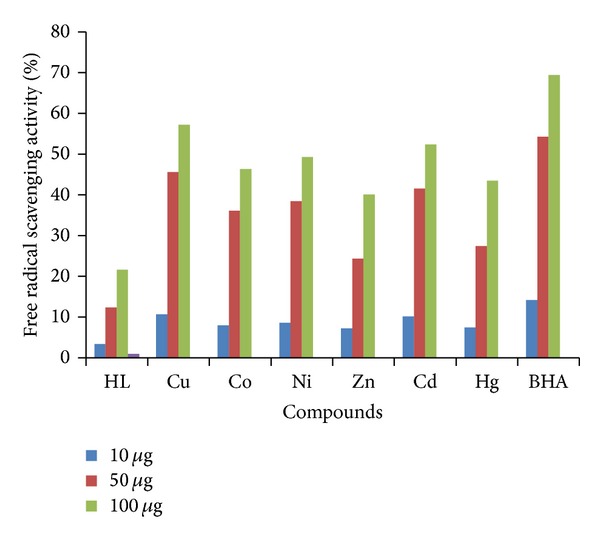
Antioxidant results of ligand and its complexes.

The newly synthesized Schiff base and its metal complexes were screened for free radical scavenging activity by DPPH method. Antioxidant activity of these compounds was investigated by measuring radical scavenging effect of DPPH radicals. The results of the free radical scavenging activity of the compounds at different concentrations are shown in Figure 6. It is evident from the results that the free radical scavenging activity of these compounds was concentration dependent. Among the examined compounds Cu(II), Cd(II), Ni(II), and Co(II) complexes have exhibited good scavenging activity. Whereas, Hg(II) and Zn(II) have shown moderate activity. All the metal complexes have exhibited higher scavenging activity than the Schiff base **HL**. The marked antioxidant activity of the metal complexes, in comparison to free Schiff base **HL**, could be due to the coordination of metal with azomethine nitrogen, carbonyl oxygen of amide function attached to the 2-position of indole, and oxygen of enolized amide function of quinolinone moiety vai deprotonation. In case of the above test compounds, the hydrogen of azomethine is more acidic than the hydrogen of the indole NH. Hence, hydrogen of azomethine could be easily donated to the DPPH free radical and convert itself into the stable free radical.

## 4. Conclusions

**Figure 7:**
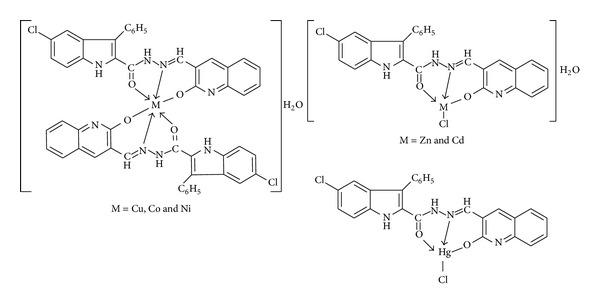
Proposed structures of metal complexes.

On the basis of the above results the Schiff base **HL** acts as tridentate (ONO) chelating agent coordinate with metal ions through the carbonyl group of amide function attached to 2-position of indole, azomethine nitrogen, and oxygen atom of the enolized amide function of quinolinone moiety via deprotonation. Analytical, spectral, and magnetic studies revealed mononuclear nature of the complexes. The probable structures of the metal complexes are shown in Figure 7. The Cu(II), Co(II), and Ni(II) complexes exhibited octahedral geometry whereas Zn(II), Cd(II), and Hg(II) complexes exhibited tetrahedral geometry. The antimicrobial activity of the Schiff base **HL** is enhanced upon complexation with metal ions particularly Cu(II), Co(II), and Ni(II) showed promising activity compared to Schiff base and proved to be essential for the growth-inhibitor effect. The DNA cleavage studies revealed that the Cu(II) complex has shown complete cleavage of genomic DNA of *E. coli*. The results of antioxidant activity experiment clearly indicate that among the test compounds Cu(II), Cd(II), Ni(II), and Co(II) complexes have exhibited good scavenging activity compared to the standard. 

## Figures and Tables

**Table 1:** Physical, analytical, magnetic susceptibility, and molar conductance data of ligand **HL** and its complexes.

Compounds	Molecular formula	Mol. Wt.	M.P (°C) (Yield in %)	Elemental analysis (%) Calcd (Found)					Mag. moment *µ*eff (B.M)	Molar cond (*µ*_M_) ohm^−1^ cm^2^ mol^−1^	Colour
				M	C	H	N	Cl			
** HL**	C_25_H_17_O_2_N_4_Cl	440	314 (65)	—	68.10 (68.25)	3.85 (3.91)	12.71 (12.89)	8.05 (8.12)	—	—	Yellow
Cu complex	[Cu(C_50_H_32_O_4_N_8_Cl_2_)]H_2_O	959.54	>340 (70)	6.61 (6.71)	62.46 (62.59)	3.53 (3.32)	11.66 (11.74)	7.39 (7.21)	1.79	31	Green
Co complex	[Co(C_50_H_32_O_4_N_8_Cl_2_)]H_2_O	954.93	>340 (77)	6.16 (6.26)	62.76 (62.92)	3.55 (3.82)	11.71 (11.86)	7.42 (7.62)	5.01	24	Brown
Ni complex	[Ni(C_50_H_32_O_4_N_8_Cl_2_)]H_2_O	954.69	>340 (79)	6.14 (6.19)	62.78 (62.92)	3.55 (3.69)	11.71 (11.91)	7.42 (7.59)	2.90	27	Brown
Zn complex	[Zn(C_25_H_16_O_2_N_4_Cl) (Cl)]H_2_O	557.40	>340 (69)	11.71 (11.62)	53.72 (53.99)	3.22 (3.35)	10.02 (10.19)	12.71 (12.89)	—	49	Light yellow
Cd complex	[Cd(C_25_H_16_O_2_N_4_Cl) (Cl)]H_2_O	604.41	>340 (71)	18.56 (18.65)	49.55 (49.71)	2.97 (2.81)	9.24 (9.35)	11.72 (11.90)	—	60	Pale yellow
Hg complex	[Hg(C_25_H_16_O_2_N_4_Cl) (Cl)]	674.59	330 (68)	29.69 (29.75)	44.40 (44.61)	2.36 (2.42)	8.28 (8.36)	10.50 (10.66)	—	50	Light Yellow

**HL **= ligand.

**Table 2:** The IR data of ligand **HL** and its complexes (cm^−1^).

Compounds	*ν*_NH/NH_	*ν*_C=O_	*ν*_H_2_O_	*ν*_C=N_	Pyridine ring	*ν*_M–O_	*ν*_M–N_	*ν*_M–Cl_
**HL**	3311, 3229, 3162	1674, 1661	—	1608	—	—	—	—
Cu complex	3283, 3221	1639, —	3418	1548	1493, 1077, 615	514	442	—
Co complex	3271, 3230	1629, —	3423	1551	1489, 1061, 670	553	435	—
Ni complex	3305, 3218	1638, —	3430	1555	1478, 1063, 621	477	450	—
Zn complex	3305, 3218	1634, —	3420	1557	1489, 1066, 615	470	425	357
Cd complex	3293, 3221	1637, —	3423	1550	1425, 1059, 699	469	419	350
Hg complex	3295, 3230	1664, —	—	1560	1420, 1060, 640	550	428	352

**HL **= ligand.

**Table 3:** The ^1^H NMR data of ligand **HL** and its Zn(II) and Cd(II) complexes.

Ligand/complexes	^ 1^H NMR data (*δ*)
**HL**	12.20 and 12.00 (s, 2H, two CONH), 11.60 (s, 1H, indole NH), 8.40 (s, 1H, HC=N), 7.10–8.20 (m, 13H, ArH).
Zn complex	13.12 (s, 1H, CONH), 11.53 (s, 1H, indole NH), 8.45 (s, 1H, HC=N), 7.35–8.55 (m, 12H, ArH).
Cd complex	13.14 (s, 1H, CONH), 11.62 (s, 1H, indole NH), 8.46 (s, 1H, HC=N), 7.20–8.35 (m, 12H, ArH).

**HL **= ligand.

**Table 4:** Electronic spectral bands and ligand field parameters of the Co(II), Ni(II), and Cu(II) complexes in DMF (10^−3^ M) solution.

Complexes	Transitions in cm^−1^			Dq (cm^−1^)	*B*′ (cm^−1^)	*β*	*β*%	*ν*_2_/*ν*_1_	LFSE (k cal)
	*ν*_1_*	*ν*_2_	*ν*_3_						
Cu(II) complex		13769–17463		1530	—	—	—	—	26.22
Co(II) complex	7447	15977	19518	853	877	0.843	9.680	1.480	14.622
Ni(II) complex	9680	15604	25634	968	812	0.780	21.92	1.470	33.188

*Calculated values.

**Table 5:** Thermal decomposition of the complexes.

Complex number	Decomposition temp (°C)	% Weight loss		Metal oxide %		Inference
		Obsd	Cald	Obsd	Cald	
Cu complex	100	1.51	1.87	—	—	Loss of lattice water molecule
	335	61.55	59.47	—	—	Loss due to two indole (2C_15_H_10_N_3_OCl) species
	519	27.88	27.87	—	—	Loss due to 2C_4_H_4_
	Upto 750	—	—	8.28	8.10	Loss due to remaining organic moiety

Co complex	100	1.88	1.59	—	—	Loss of lattice water molecule
	348	61.55	59.47	—	—	Loss due to two indole (2C_15_H_10_N_3_OCl) species
	Upto 750	—	—	8.19	8.05	Loss due to remaining organic moiety

Zn complex	110	3.09	3.22	—	—	Loss of lattice water molecule
	340	48.15	48.25	—	—	Loss due to C_15_H_10_N_2_OCl
	Upto 750	—	—	14.96	14.29	Loss due to organic moiety

Hg complex	330	5.31	5.33	—	—	Loss due to HCl
	354	49.46	48.46	—	—	Loss due to C_19_H_13_ONCl species
	Upto 850	—	—	32.25	32.09	Loss due to remaining organic moiety

**Table 6:** Powder X-ray diffraction data of Ni(II) complex.

Peak	2*θ*	*θ*	Sin⁡*θ*	Sin^2^⁡*θ*	*h* ^2^ + *k* ^2^ + *l* ^2^ (Sin^2^⁡*θ*/CF)	*h k l *	*d*		*a* in Å
							Cald.	Obsd.	
1	6.086	3.043	0.0530	0.00281	1.000 (1)	1 0 0	14.5333	14.53	14.53
2	7.385	3.692	0.0643	0.00414	1.473 (1)	1 1 0	11.9797	14.53	14.53
3	11.123	5.561	0.0969	0.00939	3.341 (3)	1 1 1	7.9494	14.53	14.53
4	12.964	6.482	0.1128	0.01274	4.533 (5)	2 1 0	6.8289	14.53	14.53
5	14.863	7.431	0.1293	0.01672	5.950 (6)	2 1 1	5.9574	14.53	14.53
6	19.509	9.754	0.1694	0.02870	10.213 (10)	3 1 0	4.5472	14.53	14.53
7	23.902	11.951	0.2070	0.04288	15.259 (15)	—	3.7212	14.53	14.53
8	25.570	12.785	0.2212	0.04897	17.427 (17)	3 2 2	3.4823	14.53	14.53
9	38.460	19.230	0.3293	0.10847	38.601 (39)	—	2.3392	14.53	14.53
10	42.710	21.355	0.3641	0.13260	47.188 (47)	—	2.1156	14.53	14.53
11	57.670	28.835	0.4822	0.23260	82.775 (83)	—	1.5974	14.53	14.53
12	60.383	30.191	0.5028	0.25289	90.000 (90)	7 5 4	1.5320	14.53	14.53
13	67.305	33.652	0.5541	0.30707	109.27 (109)	—	1.3901	14.53	14.53
14	77.026	38.513	0.6226	0.38774	137.98 (138)	8 7 5	1.2372	14.53	14.53

**Table 7:** Powder X-ray diffraction data of Zn(II) complex.

Peak	2*θ*	*θ*	Sin⁡*θ*	Sin^2^⁡*θ*	*h* ^2^ + *k* ^2^ + *l* ^2^ (Sin^2^⁡*θ*/CF)	*h k l *	*d *		*a* in Å
							Cald.	Obsd.	
1	6.953	3.4765	0.0606	0.00367	1.000 (1)	1 0 0	12.7112	12.7028	12.71
2	7.786	3.8930	0.0678	0.00460	1.253 (1)	1 0 0	11.3613	11.3461	12.71
3	10.094	5.0470	0.0879	0.00739	2.013 (2)	1 1 0	8.7633	8.7561	12.71
4	12.115	6.0575	0.1055	0.01113	3.032 (3)	1 1 1	7.3014	7.2995	12.71
5	14.757	7.3785	0.1284	0.01649	4.493 (4)	2 0 0	5.9992	5.998	12.71
6	16.104	8.0522	0.1400	0.01962	5.346 (5)	2 1 0	5.5021	5.499	12.71
7	18.325	9.1625	0.1592	0.02535	6.907 (7)	—	4.8385	4.8374	12.71
8	20.999	10.199	0.1770	0.03135	8.542 (9)	2 2 1, 3 0 0	4.3519	4.3501	12.71
9	23.474	11.737	0.2034	0.04137	11.272 (11)	3 1 1	3.7871	3.7867	12.71
10	25.247	12.623	0.2185	0.04776	13.013 (13)	3 2 0	3.5254	3.5247	12.71
11	32.675	16.3375	0.2812	0.07912	21.558 (22)	3 3 2	2.7393	2.7384	12.71
12	38.105	19.052	0.3264	0.1065	29.032 (29)	5 2 0	2.3599	2.3597	12.71
13	45.866	22.933	0.3896	0.1518	41.370 (41)	6 2 1	1.9771	1.9768	12.71
14	48.416	24.208	0.4100	0.1681	45.814 (46)	6 3 1	1.8780	1.8785	12.71
15	61.823	30.911	0.5137	0.2639	71.907 (72)	6 6 0	1.4995	1.4994	12.71

**Table 8:** Minimum inhibitory concentration (MIC *µ*g mL^−1^) of ligand and its metal complexes.

Compounds	MIC value in *µ*g mL^−1 ^(zone of inhibition in mm)						
	*E. coli *	*S. aureus *	*B. subtilis *	*S. typhi *	*C. albicans *	*C. oxysporum *	*A. niger *
**HL**	50	75	50	100	75	50	50
Cu complex	12.50	25	25	50	25	12.50	12.50
Co complex	25	50	12.50	50	12.50	25	25
Ni complex	12.50	25	25	50	25	12.50	25
Zn complex	25	50	25	75	50	25	25
Cd complex	12.50	12.50	12.50	25	12.50	25	12.50
Hg complex	25	12.50	25	50	25	25	12.50
Gentamycin	12.50	12.50	12.50	12.50	—	—	—
Fluconazole	—	—	—	—	12.50	12.50	12.50

**HL** = ligand.
